# Love Is Blind: Indiscriminate Female Mating Responses to Male Courtship Pheromones in Newts (Salamandridae)

**DOI:** 10.1371/journal.pone.0056538

**Published:** 2013-02-15

**Authors:** Dag Treer, Ines Van Bocxlaer, Severine Matthijs, Dimitri Du Four, Sunita Janssenswillen, Bert Willaert, Franky Bossuyt

**Affiliations:** Amphibian Evolution Lab, Vrije Universiteit Brussel, Brussels, Belgium; CNRS, France

## Abstract

Internal fertilization without copulation or prolonged physical contact is a rare reproductive mode among vertebrates. In many newts (Salamandridae), the male deposits a spermatophore on the substrate in the water, which the female subsequently takes up with her cloaca. Because such an insemination requires intense coordination of both sexes, male newts have evolved a courtship display, essentially consisting of sending pheromones under water by tail-fanning towards their potential partner. Behavioral experiments until now mostly focused on an attractant function, i.e. showing that olfactory cues are able to bring both sexes together. However, since males start their display only after an initial contact phase, courtship pheromones are expected to have an alternative function. Here we developed a series of intraspecific and interspecific two-female experiments with alpine newt (*Ichthyosaura alpestris*) and palmate newt (*Lissotriton helveticus*) females, comparing behavior in male courtship water and control water. We show that male olfactory cues emitted during tail-fanning are pheromones that can induce all typical features of natural female mating behavior. Interestingly, females exposed to male pheromones of their own species show indiscriminate mating responses to conspecific and heterospecific females, indicating that visual cues are subordinate to olfactory cues during courtship.

## Introduction

Internal fertilization without copulation or another form of prolonged physical contact is a rare reproductive mode among vertebrates. In many newts (Salamandridae), the male deposits a spermatophore (a gelatinous mass topped with a packet of sperm) on the substrate in the water, which the female subsequently takes up with her cloaca. Because successful insemination in these animals requires intense coordination of both sexes, males have evolved a courtship display with several variations of tail-waving (henceforth used for different types of tail movements, but essentially consisting of tail-fanning, i.e. tail folded against the male's flank while undulating fast) towards the female's snout [1]–[4] (Movie S1). A female reacts by closely following the courting male [5]–[7] (Movie S1). The male then turns away from the female and she touches his tail, thereby confirming her following and prompting spermatophore deposition [1] (Movie S1). The whole courtship sequence ends with the female being lead over the spermatophore, which adheres to her cloaca and results in insemination. Although it is obvious that male tail-waving plays a central role in the courting process, and despite this behavior was initially described already two centuries ago [8], [9], the exact effects of male tail-waving on female behavior remain unknown.

The involvement of both visual and olfactory stimuli during courtship has been proposed. First, males develop species-specific epigamic characters during the breeding season, such as skin extensions (e.g. on legs, tail and crest) and intense coloration patterns [2]. The impact of these visual stimuli during courtship is poorly understood [1], [10], but some studies suggested a direct or indirect female sexual preference for some of these male visual traits [11]–[15]. Second, males have sexually dimorphic glands in their cloaca [16], [17], and it is generally acknowledged that male tail-fanning helps to send pheromones from the male's cloaca towards the snout of the female [3], [18]–[21]. This is also obvious from the fact that the male opens his cloaca during tail-waving (personal observation).

Known sex pheromones in salamanders can be categorized in two main groups. Attractant pheromones facilitate the location of potential mates and serve to bring males and females together [22]. Conversely, courtship pheromones are used only after initial contact with a potential mate, and operate by altering female behavior during courtship [23], [24]. Until now, only one population-specific decapeptide [25] and its two species-specific variants [26] in the genus *Cynops* have been described in salamandrids. Using experiments with sponges [3], [25]–[27], these molecules were shown to work as sex attractants. In other salamandrids, experiments with linear olfactometers [28], Y-mazes [21], [29] and two-choice aquaria [30], [31] also suggest the presence of attractant pheromones. However, since a male only starts tail-waving in front of the female after an initial investigation [1], [2], [4], [5], [32], the pheromones emitted during this behavior are expected to be courtship rather than attractant pheromones [23], [24], and a straightforward function for these chemical cues therefore remains unknown.

The demonstration and functional interpretation of courtship pheromones preferentially requires a behavioral experiment that, in addition to control over pheromone presence, includes the visual and tactile stimuli of a second animal mimicking the presence of a mating partner in a natural courtship. Here we developed such experiments with two females. Using a second female instead of a male allowed us to separate the male's visual secondary sexual characters and his courtship display from olfactory cues. To test for a pheromone function, we used water in which a male had been courting another male as a source of olfactory cues. We show that the male chemical cues emitted during tail-waving are courtship pheromones that induce all typical features of natural female responses culminating in spermatophore pick-up from the substrate. Because this induced female behavior is essential in getting her cloaca positioned for spermatophore pick-up, these pheromones are a key aspect of insemination without physical contact in salamandrids.

## Materials and Methods

### Ethics statement

The research was done with permission and according to the guidelines of Agentschap voor Natuur en Bos (permit ANB/BL-FF/V12-00050). All experiments complied with EU and Belgian regulations concerning animal welfare. Animals were released back to the pond of their origin after the experiments were finished.

### Animals

For both alpine newts (*Ichthyosaura alpestris*) and palmate newts (*Lissotriton helveticus*), 40 adult females and 40 adult males were collected in February/March 2012 from a pond near Hasselt, Belgium, using a modified Ortmann's funnel trap [33]. They were housed in single sex aquaria (60×35×35 cm) filled up with 30 liters of aged tap water and containing vegetation from the pond of the newts' origin. Up to 15 newts were kept together per aquarium. The temperature and light regime were artificially regulated (15–18°C, 13 h/11 h light/dark). Newts were fed *ad lib* with maggots or earthworms every other day.

### Receptivity test

Behavioral experiments and collection of stimuli were preceded by a receptivity test on the day before the experiment, and only receptive animals were selected. Receptivity was tested by putting a male and a female together in a plastic container (25×16×14 cm) filled with 800 ml of aged tap water. Males that courted successfully were considered receptive, and were used for collection of chemical stimuli. Only females that followed a courting male were selected for the behavioral experiments. During the receptivity test, females were not allowed to pick up spermatophores.

### Chemical stimuli and control

Collection of male courtship water was done by putting two receptive males in a plastic container (25×16×14 cm) filled with 800 ml of aged tap water. To ensure similar concentrations in all experiments, we measured ten minutes of active male tail-fanning. Approximately one on eight males was tail-fanning to another male for this amount of time. The males were then removed and the male courtship water was used immediately in the behavioral experiment. The water of two non-courting males was collected in the same way and for the same time as above, but for each male separately to ensure that there was no courtship display. We also performed experiments using this water to show that the observed courtship behavior is induced by molecules that are released during male courtship display only. Control behavioral experiments were done in aged tap water. For all experiments, we used aged tap water that had been kept in the room where the animals were housed, ensuring similar temperature conditions.

### Experiment design

Experiments were conducted in a plastic container (25×16×14 cm) covered with brown cardboard from the outside to prevent outside visual stimuli. The container was filled with 800 ml of either chemical stimuli or control water. Two randomly picked receptive females were taken with a glove from their housing container, briefly rinsed in aged tap water and placed in the experiment container. Their behavior was recorded for 10′ using a digital camera connected directly to a computer, where the video recordings were stored for later analyses. Females were used in one experiment per day only and only once in each experimental design. To reduce potential variation in receptivity to a minimum, all experiments were performed on consecutive days. They were done during the day, at the same time of the day, and under the same light and temperature conditions. We performed both intraspecific (using two alpine newt females) and interspecific experiments (using one alpine newt and one palmate newt female).

### Data analysis and statistics

**Figure 1:**
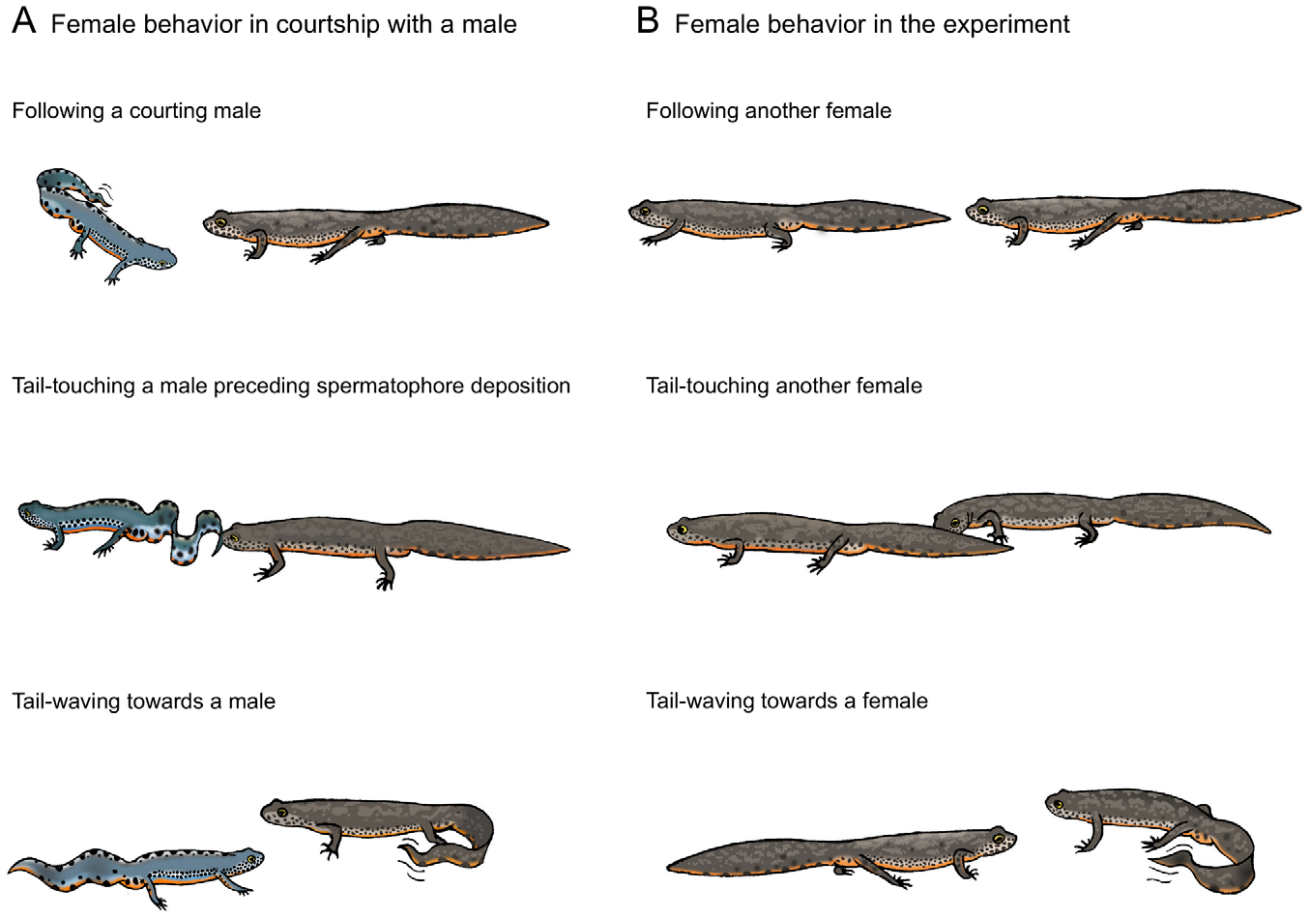
Comparison of natural and experimental mating behavior. (**A**) *Female natural mating behavior:* the female follows the tail-waving male, touches the male's tail to stimulate spermatophore deposition, and uses tail-waving to encourage a male to continue courtship (see Materials and methods). (**B**) *Equivalent female behavior in a two-female experiment:* after addition of male courtship water, one female follows the other one, or both females try to follow each other; the following female regularly touches the tail of the other one; a female uses tail-waving in trying to encourage the other female.

The video recordings of the experiments were analyzed for female responses similar to those under natural conditions with a male. For statistical comparison, we classified behavior in three quantifiable components (Fig. 1): First, females showed the typical following behavior (*f*), i.e. they closely followed the movements of the other female. Second, the following female frequently touched the tail (*tt*) of the leading female, thereby mimicking the natural behavior of tail-touching before spermatophore deposition. Third, male courtship water eventually induced female tail-waving (*w*) behavior (resembling that of male tail-fanning), similar to that shown after prolonged courtship with a male. These behaviors can be shown by one of the two females, but sometimes both females tried to follow each other simultaneously, resulting in a wheel-shaped movement, or reciprocal tail-waving. Quantification was done as follows:

1. *Following*: the amount of time in which a female incessantly shows interest towards the other female, including turning towards another female and following her. Because this could be considered objective, we also measured pointing, i.e. the amount of time that an imaginary straight line, perpendicular to the line connecting the eyes of the following female, intersects the other female's body. The results of these analyses (not shown) were very similar and equally statistically significant.
2. *Tail-touching*: the duration of a female touching another female's tail with her snout. To avoid interpreting accidental touching (e.g., as the result of the first female moving her tail against the following female) as a positive response, only the female's snout actively pressed against the other female's tail at an angle of 45°–90° was measured.
3. *Tail-waving*: the duration of one female waving her tail towards the other female (see results). It was measured from the time the female starts bending her tail to start waving until the tail was bent back more than 90° away from the female's body.

The cumulative duration of each behavior was measured in seconds. The differences in behavior between different stimuli were tested with the Kruskal-Wallis test followed by *post hoc* two-tailed Mann-Whitney U test [34] using SPSS [35].

## Results

### Female tail-waving to a male

Although tail-waving is often regarded as a male-specific behavior, we observed this behavior in females during receptivity tests. Females were not allowed to pick-up spermatophores during these tests, which resulted in prolonged courtship and eventually reduced male courtship display. In many instances, the female then started tail-waving to the male (Movie S1), but stopped this behavior and continued following as soon as the male resumed his courtship display. This female tail-waving was only observed after an initial period of male courtship, i.e., after a female had already been following a male, indicating that this behavior is performed only after contact with pheromones. Although the function of male pheromones is not to induce female tail-waving, this behavior occurs in male courtship water, but not in non-courting male water and control water, and therefore was used in our experiments as indirect evidence of active courtship pheromones.

### Intraspecific two-female experiments in alpine newts

**Figure 2:**
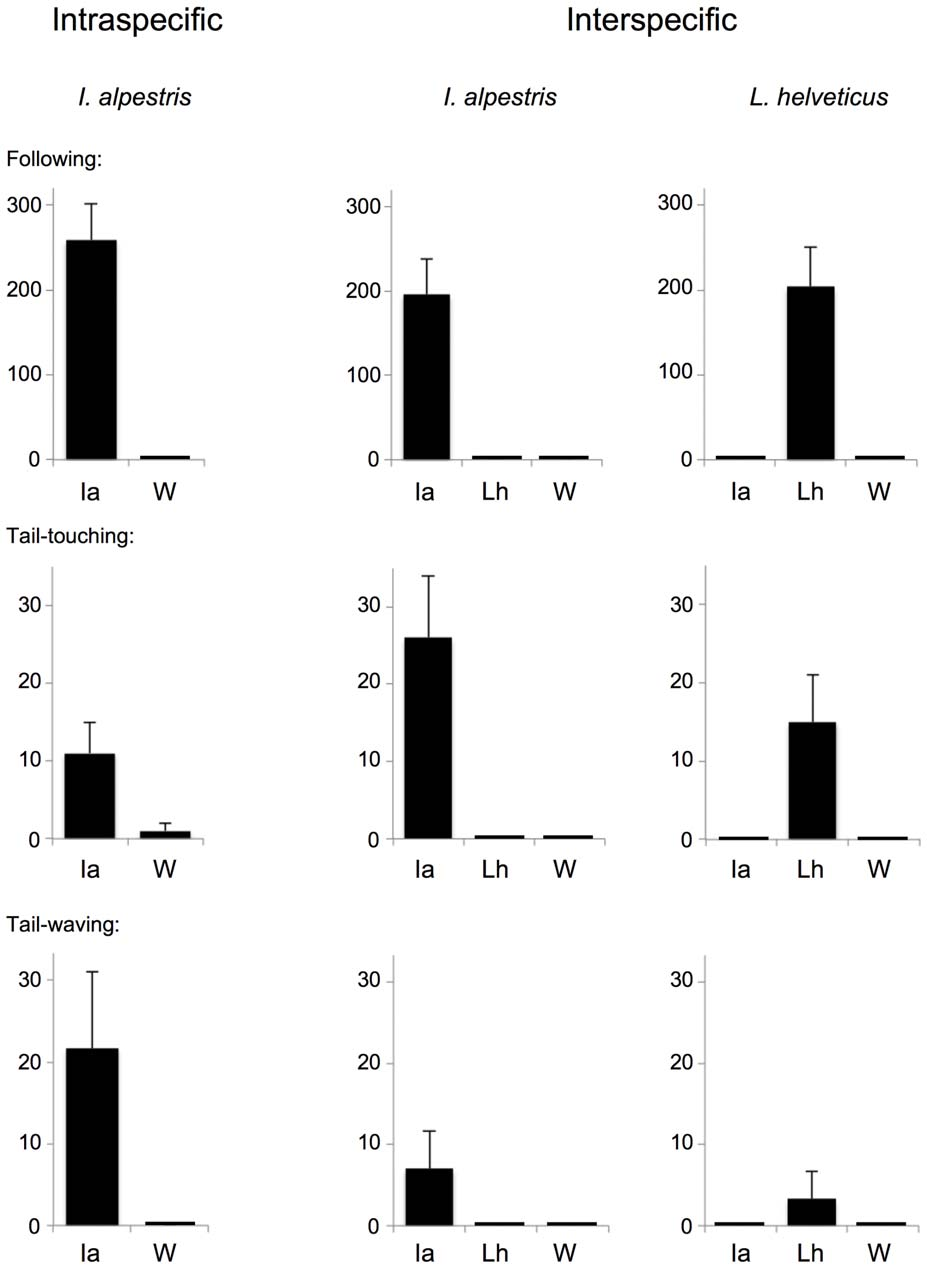
Results of intra-specific and inter-specific two-female experiments. The experiments show that the pheromones are species-specific and extremely potent. The focal species in the experiment is indicated on top. The mean cumulative duration of the behaviour in seconds (+/− S.E.) is indicated on the y-axis. Abbreviation for stimuli (indicated on x-axis): Ia, alpine newt male courtship water; Lh, palmate newt male courtship water; W, control water.

Our two-female experiment with two alpine newts showed that male courtship water induces following, tail-touching and tail-waving in females (Movie S2), while these behaviors are practically absent in control water (two-tailed Mann-Whitney U-test, *P_f_*<0.001, *P_tt_*<0.001 and *P_w_*<0.05, respectively) (Fig. 2). All typical features of a female's mating responses can thus be evoked without the male's visual secondary sexual and behavioral characteristics, such as the crest and coloration, and tail-waving, respectively. Tests with water in which non-courting males had been kept were not significantly different from control water (*P_f_* = 0.152; *P_tt_* = 0.130, *P_w_* = 0.317), but were significantly different from male courtship water (*P_f_*<0.001; *P_tt_*<0.05, *P_w_*<0.01) (Table 1). Altogether, these experiments indicate that the female behavioral responses in alpine newts are caused by pheromones emitted during the male's courtship display, and do not require male-specific visual stimuli.

**Table 1:** Results of the statistical tests.

stimulus 1	*N*	stimulus 2	*N*	focal female	behavior	Mann-Whitney U	*U*	*Z*
Intraspecific test (*I. alpestris*+*I. alpestris*)								
Ia	20	MW	20	*I. alpestris*	f	<0.001*	*42.000*	*−4.621*
					tt	<0.05*	*123.000*	*−2.207*
					w	<0.01*	*126.500*	*−2.719*
Ia	20	W	20	*I. alpestris*	f	<0.001*	*30.000*	*−5.109*
					tt	<0.001*	*93.000*	*−3.269*
					w	<0.05*	*120.000*	*−3.097*
MW	20	W	20	*I. alpestris*	f	0.152	*180.000*	*−1.432*
					tt	0.130	*156.000*	*−1.514*
					w	0.317	*190.000*	*−1.000*
Interspecific test (*I. alpestris*+*L. helveticus*)								
Ia	15	W	15	*I. alpestris*	f	<0.001*	*90.000*	*−3.782*
					tt	<0.001*	*22.500*	*−4.215*
					w	<0.05*	*75.000*	*−2.396*
Lh	15	W	15	*I. alpestris*	f	1.000	*112.500*	*0.000*
					tt	0.150	*97.500*	*−1.438*
					w	1.000	*112.500*	*0.000*
Interspecific test (*I. alpestris*+*L. helveticus*)								
Ia	15	W	15	*L. helveticus*	f	1.000	*112.500*	*0.000*
					tt	0.524	*104.500*	*−0.637*
					w	1.000	*112.500*	*0.000*
Lh	15	W	15	*L. helveticus*	f	<0.001*	*37.500*	*−3.707*
					tt	<0.05*	*69.000*	*−2.225*
					w	0.317	*105.000*	*−1.000*
Intraspecific vs. interspecific female preference								
Ia (+*I. alpestris*)	20	Ia (+*L. helveticus*)	15	*I. alpestris*	f	0.340	*121.500*	*−0.954*
					tt	0.183	*110.500*	*−1.333*
					w	0.398	*128.000*	*−0.846*

Ia = alpine newt male courtship water, MW = water in which non-courting alpine newt males had been kept, W = control water, Lh = palmate newt male courtship water, N = number of different females used in the experimental design. *P*-values smaller than 0.05 are considered significant and are indicated with an asterisk. The values for Kruskal-Wallis tests were as follows: intraspecific tests (*P_f_*<0.05, *P_tt_*<0.05 and *P_w_*<0.05), interspecific tests for alpine newt females (*P_f_*<0.05, *P_tt_*<0.05 and *P_w_*<0.05), interspecific tests for palmate newt females (*P_f_*<0.05, *P_tt_*<0.05 and *P_w_* = 0.096).

### Interspecific experiments

To further test the requirement of conspecific (but not necessarily male-specific) visual cues and species-specificity of male courtship chemical stimuli, we repeated our two-female experiment using one alpine newt and one palmate newt. The latter species is much smaller and lacks the orange belly and dark dorsal skin (species-specific visual stimuli in alpine newts [36]), but shows similar courtship behavior [37]. Our experiments indicate that alpine newt male courtship water elicited the three typical courtship behaviors in alpine newt females (*P_f_*<0.001; *P_tt_*<0.001, *P_w_*<0.05) but not in palmate newt females (*P_f_* = 1; *P_tt_* = 0.524; *P_w_* = 1), while the opposite was true in palmate newt male courtship water (palmate newt females: *P_f_*<0.001; *P_tt_*<0.05, *P_w_* = 0.317; alpine newt females *P_f_* = 1; *P_tt_* = 0.150; *P_w_* = 1) (Fig. 2) (Movie S3). These experiments indicate that male courtship chemical stimuli are species-specific, because they only stimulate conspecific females while the other species' behavior remains unaffected and similar to that in control water. Furthermore, affected females of alpine newts found females of both species equally seductive in alpine newt male courtship water (*P_f_* = 0.340; *P_tt_* = 0.183; *P_w_* = 0.398) (Table 1), indicating that chemical stimuli can equally induce female courtship behavior towards another newt, i.e. in the absence of species-specific and gender-specific visual cues.

## Discussion

Behavioral studies searching for pheromones in Salamandridae until now had focused on an attractant function of the chemical stimuli, i.e. showing that olfactory communication brings males and females together. However, although such experiments indicated olfactory recognition in salamandrids, they did not provide a satisfactory explanation for one of the most obvious behaviors indicating pheromone use, i.e. male tail-fanning. Our two-female experiments here show that pheromones emitted during male courtship are able to induce all female responses that precede spermatophore pick-up. This is evidenced by three behaviors, following, tail-touching and tail-waving, all of which can be observed under influence of male pheromones released during tail-fanning, but are basically non-existent in the absence of these molecules. The olfactory cues emitted during male tail-waving therefore resort under the group of courtship pheromones, i.e. molecules aimed at changing behavior in females. Male newts that perform courtship more energetically are known to have a higher mating success [38], which in light of our experiments can be explained as a more efficient transfer of olfactory stimuli.

Our interspecific experiments suggest that visual cues are largely irrelevant when the pheromone has reached a certain threshold in the female. This helps to explain the occurrence of sexual interference in newts, i.e., males interfering in courtship of another male. During courtship, a second male sometimes intervenes between both sexes and deposits a spermatophore in front of the already following female [6], [39], [40]. We hypothesize that such newts exploit olfactory stimuli of another male and the indiscriminate behavior of the following female to enhance their own reproductive success. Male visual stimuli would then mainly serve as sexual defense against such sexual interference [41]. Being more visually attractive would insure a courting male that he remains the main focus of the female that is stimulated by olfactory stimuli [1], [42]. A higher importance of olfactory stimuli over visual stimuli had already been shown in experiments with high light and low light conditions in alpine newts [43]: Although both sexes spent significantly more time in orientation (without courtship displays) in low light conditions, males were performing more tail-fanning and obtained more positive response from females, resulting in similar mating success as in high light conditions.

Our experiments surprisingly revealed that females under influence of male courtship pheromones can show tail-waving to a potential mating partner, a behavior that is suggestive of the presence of female olfactory cues. This kind of male-like behavior has been termed ‘pseudo-male’ or ‘heterotypical’ female behavior [44]–[47] and might be caused by sexual frustration, when females are deprived of males and are highly receptive [44], [46]. We therefore hypothesize that, just as in males, tail-waving helps the female to deliver her olfactory stimuli [48], [49] more efficiently to the male, thereby stimulating him to continue courtship. Olfactory investigation of females by males is common prior and during courtship [1], [2], [4], [5], [23], [32]. For example, female olfactory cues are known to induce male tail-fanning in the smooth newt [50]. Additionally, crested newt males do not show sexual behavior after their nostrils are plugged and female olfactory cues cannot reach them [51].

The inferred fundamental role of pheromone use in newts has important implications for their reproduction. If the female does not follow the courting male, he will rarely deposit a spermatophore, and even if he would, chances that it would be picked up by the female are low [1], [5], [7], [10]. Therefore, male courtship pheromones in salamandrids are a necessary prerequisite for newt reproduction. Such a strong dependence on a pheromone is unusual in amphibians. In lungless salamanders (Plethodontidae), it has been shown that two pheromones (SPF and PRF), alone or in combination with another pheromone (PMF), reduce courtship time (i.e., the time to spermatophore deposition) [52]–[54]. However, spermatophore deposition in this family equally occurs in salamanders after ablation of their courtship mental gland, i.e. in the absence of these pheromones. Since this is not the case in salamandrids [20], [55], such a strong olfactory dependence would become critical for newt survival if pollution would interact directly (e.g., chemical interaction with the pheromones in the water) or indirectly (e.g., by altering hormone levels involved in the pathway of pheromone production) with the pheromone system [56], [57].

Our behavioral experiments indicate that the pheromone function of tail-waving is evolutionary conserved in at least two lineages, *Ichthyosaura* and *Lissotriton*. Variations aside, a courtship that basically consists of tail-waving and the male leading the female over the deposited spermatophore is present in multiple genera and species of newts [1], [2], [4], [5], [32]. It is therefore likely that this pheromone function originated early in newt evolution and has been retained in several genera. Similar to pheromones in Plethodontidae, salamandrid pheromones are likely to constitute fast evolving molecules [58]–[61]. Our two-female experiments indicate that the olfactory cues produced during tail-waving are species-specific (hence the term pheromone can be used, [62]) only inducing the typical behavior in females of the same species. These molecules therefore have the potential to function as a significant mechanism of reproductive isolation [26], [31], [63]. Multiple species of newts co-inhabit the same ponds during the breeding season [64], and their crepuscular or nocturnal lifestyle [65], [66] in often turbid water [67] can make visual mate recognition difficult. The recognition of conspecific male courtship pheromones is probably an important mechanism in maintaining reproductive isolation and reducing hybridization in salamandrids.

## Conclusions

Interpretation of the function of a pheromone often depends on the behavioral experiments being performed. Although it could be argued that pheromones produced during tail-waving are attractants because they keep the female close to the male due to following, they essentially are courtship pheromones, i.e. they alter female courtship responses [24]. Our two-females experiments reveal that male courtship pheromones are extremely potent, rendering even females of other genera irresistible to exposed females. The essence of male courtship pheromones therefore goes beyond the traditionally tested attractant function, and is an essential element for eliciting the mating behavior in females leading to successful insemination without physical contact. Our new behavioral experiment, that seems to work over a broad range of species, opens possibilities for the identification and study of pheromones across the whole clade of newts.

## Supporting Information

Movie S1 **Male-female natural mating behavior in alpine newts.**
(MP4)

Movie S2 **Two-female experiments (intraspecific).**
(MP4)

Movie S3 **Two-female experiments (interspecific).**
(MP4)
